# LBH589 Inhibits proliferation and metastasis of hepatocellular carcinoma via inhibition of gankyrin/stat3/akt pathway

**DOI:** 10.1186/1476-4598-12-114

**Published:** 2013-10-05

**Authors:** Xuan Song, Jiabei Wang, Tongsen Zheng, Ruipeng Song, Yingjian Liang, Nishant Bhatta, Dalong Yin, Shangha Pan, Jiaren Liu, Hongchi Jiang, Lianxin Liu

**Affiliations:** 1 Department of Hepatic Surgery, The First Affiliated Hospital of Harbin Medical University, Key Laboratory of Hepatosplenic Surgery, Ministry of Education, 150001 Harbin, Heilongjiang Province, China; 2 Harvard Medical School, 300 Longwood Avenue, 02115 Boston, MA, USA; 3 Key Laboratory of Hepatosplenic Surgery, Ministry of Education, Department of General Surgery, The First Affiliated Hospital of Harbin Medical University, No. 23 Youzheng Street, 150001 Harbin, Heilongjiang Province, China

**Keywords:** Hepatocellular carcinoma, LBH589, Apoptosis, Gankyrin, STAT3

## Abstract

**Background:**

Gankyrin has shown to be overexpressed in human liver cancers and plays a complex role in hepatocarcinogenesis. Panobinostat (LBH589), a new hydroxamic acid-derived histone deacetylase inhibitor has shown promising anticancer effects recently. Here, we investigated the potential of LBH589 as a form of treatment for hepatocellular carcinoma (HCC).

**Methods:**

Gankyrin plasmid was transfected into HCC cells, and the cells were selected for more than 4 weeks by incubation with G418 for overexpression clones. The therapeutic effects of LBH589 were evaluated *in vitro* and *in vivo*. Cell proliferation, apoptosis, cell cycle, invasive potential, and epithelial-mesenchy-mal transition (EMT) were examined.

**Results:**

LBH589 significantly inhibited HCC growth and metastasis *in vitro* and *in vivo*. Western blotting analysis indicated that LBH589 could decrease the expression of gankyrin and subsequently reduced serine-phosphorylated Akt and tyrosine-phosphorylated STAT3 expression although the total Akt and STAT3 were unaffected. LBH589 inhibited metastasis *in vitro* via down-regulation of N-cadherin, vimentin, TWIST1, VEGF and up-regulation of E-cadherin. LBH589 also induced apoptosis and G1 phase arrest in HCC cell lines. Ectopic expression of gankyrin attenuated the effects of LBH589, which indicates that gankyrin might play an important role in LBH589 mediated anticancer effects. Lastly*, in vivo* study indicated that LBH589 inhibited tumor growth and metastasis, without discernable adverse effects comparing to control group, with abrogating gankyrin/STAT3/Akt pathway.

**Conclusions:**

Our results suggested that LBH589 could inhibit HCC growth and metastasis through down-regulating gankyrin/STAT3/Akt pathway. LBH589 may present itself as a novel therapeutic strategy for HCC.

## Introduction

Hepatocellular carcinoma ranks among the most common malignancies in Asia and the third most frequent cause of cancer death worldwide [1,2]. Although there are several modalities of HCC treatment, most patients present with unresectable tumors, and nonsurgical treatments are minimally effective at the most. Given the grim outlook of HCC, novel therapeutic targets and new modalities of effective chemoprevention and treatment is highly awaited.

Now there is a growing evidence indicating that epigenetic and genetic changes are crucial for the onset and progression of malignant disease [3]. Many of the changes in gene expression resulting in malignant transformation result from altered epigenetic regulation including DNA methylation and histone deacetylation [4,5]. The histone deacetylase (HDAC) enzymes control the structural conformation of chromatin via deacetylation of core nucleosomal histones [6]. HDACs can close chromatin, so transcription factors cannot access DNA furthermore suppressing gene expression. In addition to histones, HDACs can modulate the function of many other proteins involved in the regulation of cell survival and proliferation, angiogenesis, inflammation, and immunity.

The deacetylase inhibitors are a structurally diverse class of targeted anticancer agents that have shown *in vitro* and *in vivo* preclinical activity. Among these, the deacetylase inhibitor panobinostat, (LBH589, Novartis Pharmaceuticals, Basel, Switzerland) is the most widely studied. The extensive pharmacokinetic, pharmacodynamic and dose-findings are available for a wide variety of hematologic and solid malignancies which obviously gives superiority over others. It belongs to the structurally novel cinnamic hydroxamic acid class of compounds and is currently in clinical development for both intravenous and oral formulation [7].

Gankyrin, Fujita et al. using complementary DNA subtractive hybridization found a seven ankyrin-repeat protein [8]. It was initially characterized as an oncoprotein commonly overexpressed in hepatocellular carcinoma and independently as a protein associated with the 19S regulatory complex of the 26S proteasome. Furthermore, inhibition of gankyrin could induce apoptosis in cancer cells, especially in liver cancer cells [1]. Gankyrin gene is also one of the important genes over-expressed in a rodent model of hepatocarcinogenesis [9]. Therefore, gankyrin is a promising target for potential anti-liver cancer therapeutic agents.

Against this background, we hypothesize that LBH589 might be used as a promising modality for HCC treatment. In the present study, we sought to evaluate the therapeutic potency of LBH589 toward HCC by *in vivo* and *in vitro* experiments. We extensively investigated the function of LBH589 and determined its contribution to inhibit HCC proliferation and metastasis. We also elucidated the molecular mechanisms by which LBH589 inhibits tumor proliferation and metastasis. Results presented here suggest that gankyrin/STAT3/Akt pathway plays an important role in the treatment of LBH589. We propose that LBH589 is a new powerful chemotherapeutic for HCC.

## Materials and methods

### Cell lines and LBH589 treatment

Liver cancer cell lines SMMC-7721 and HCC-LM3 were purchased from Cell Bank of Type Culture Collection of Chinese Academy of Sciences, Shanghai Institute of Cell Biology, Chinese Academy of Sciences; HepG2 cell line was obtained from American Type Culture Collection (Manassas, VA). HCC-LM3, HepG2 and SMMC-7721 cell lines were maintained at 37°C in a humidified incubator containing 5% CO_2_, in Dulbecco’s Modified Eagle Medium supplemented with 10% fetal bovine serum and 1% penicillin/streptomycin. LBH589 was provided by Novartis Pharmaceuticals, Inc. (East Hanover, NJ). LBH589 was dissolved in DMSO (Sigma, St. Louis, MO) and stored as a 30 mmol/L stock solution in small aliquots at −20°C.

### MTT assay

HCC cells were seeded at 2 × 10^4^ per well in 96-well flat-bottomed plates and incubated in 10% FBS supplemented DMEM for 24 h. Cells were treated with LBH589 at various concentrations in the same medium. Controls received DMSO vehicle at a concentration equal to that in drug-treated cells. After 24, 48 and 72 h, the drug-containing medium was replaced with 200 μL of 10% FBS supplemented DMEM containing 0.5 mg/mL MTT, and cells were incubated in the CO_2_ incubator at 37°C for 4 h. Medium was removed, the reduced MTT was solubilized in 100 μL per well of DMSO, and measured absorbance at 570 nm.

### Plasmid construction and transfection

For gankyrin overexpression, The whole cDNA sequence of gankyrin (from pCMV-HA-gankyrin) was cloned into the pCDNA-3.1A-myc vector and obtained myc-gankyrin construct. pCMV-HA-gankyrin and pCDNA-3.1A-myc were purchased from Biowot Technologies (Shenzhen, China). Control plasmid and myc-gankyrin were transfected into HCC cells using Lipofectamine 2000 (Invitrogen, Carlsbad, CA) following the manufacturer’s protocol. The cells were selected for more than 4 weeks by incubation with G418 (Invitrogen, 400 ng/ml for SMMC-7721 and 600 ng/ml for HCC-LM3 and HepG2) for overexpression clones. Stable single clones were selected and myc expression assessed using western blotting. Transient trans fection of pCMV-HA-gankyrin and the control constructs into HCC cells were performed using Lipofectamine 2000.

### RNA interference

Gankyrin-specific shRNA, synthesized by GeneChem Corporation (Shanghai, China), was designed to silence all splices of human gankyrin mRNA. The sequence was: forward, 5′- Ccggca GGT TGG TCT CCT CTT CAT ATT CAA GAG ATA TGA AGA GGA GAC CAA CC tgTTTTTg-3′; reverse, 5′-aattcaaaaaca GGT TGG TCT CCT CTT CAT ATC TCT TGA ATA TGA AGA GGA GAC CAA CC tg-3′. It was scrambled to generate a negative control. Lentivirus vectors expressing shRNA targeting gankyrin (Lenti-shgankyrin) was constructed, packed, and purified by GeneChem Corporation (Shanghai, China).

### Cell cycle and apoptosis analysis

Cell cycle analysis kit and Annexin V–FITC apoptosis kit were purchased from Becton Dickinson, San Diego, CA. For cell cycle analysis, the cells were harvested after treatment, fixed with ice-cold 70% ethanol solution, hydrolyzed with 250 μg/ml of RNaseA at 37°C for 30 min, and stained with propidium iodide at 10 mg/ml for 20 min. We analyzed the DNA content by FACSCalibur flow cytometer (Becton Dickinson, San Diego, CA).

For apoptosis analysis, the cells were harvested after treatment, washed twice with pre-chilled PBS and resuspended in 1x binding buffer at a concentration of 1 × 10^6^ cells/ml. 100 μl of such solution (1 × 10^5^ cells) was mixed with 5 μl of Annexin V-FITC and 5 μl of propidium iodide according to the manufacturer’s instruction. The mixed solution was gently vortexed and incubated in the dark at room temperature (25°C) for 15 min. 400 μl of 1x dilution buffer was then added to each tube and cell apoptosis analysis was performed by FACSCalibur flow cytometer within 1 hour.

### Western blotting

For preparing total cell lysates, cells were lysed in lysis buffer (Invitrogen), incubated on ice for 30 min and centrifuged for 20 min to remove cell debris. Total cell lysate was subjected to SDS–polyacrylamide gel electrophoresis. The proteins were then electro-transferred to polyvinylidene difluoride membrane (Millipore, Billerica, MA) and incubated overnight with antibodies at 4°C. Subsequently, the membranes were incubated with secondary antibodies for 1 hour at room temperature and the signal was detected using an enhanced chemiluminescence detection kit (Pierce, Rockford, IL). The primary antibodies were acetyl-Histone H3, acetyl-Histone H3 (Lys27) and acetyl-Histone H4 were purchased from Millipore. Histones were isolated according to the instruction of the manufacturer. STAT3, p27, HA-tag, myc-tag, p-STAT3 (Tyr705), Akt, p-Akt (Ser473), PI3K, p-PI3K (Tyr458), JAK2, p-JAK2 (Tyr1007/1008), Bcl-xL, cleaved-PARP, cleaved-caspase-3, 8 and 9 were purchased from Cell Signaling Technology (Danvers, MA). CD31 was purchased from Novus (Littleton, CO). Ki-67, N-cadherin, E-cadherin and vimentin were purchased from Abcam (Cambridge, MA). Gankyrin, p16, p53, Rb, VEGF, cyclinD1, cyclinE, tubulin and β-actin were purchased from Santa Cruz Biotechnology (Dallas, Texas). The secondary antibodies, anti-mouse IgG-HRP and anti-rabbit IgG-HRP were purchased from Santa Cruz Biotechnology.

### Interleukin-6 (IL-6) determination

Detection and quantitative measurement of human IL-6 in cell culture supernatants were performed by the Human IL-6 ELISA kit (R&D Systems, Minneapolis, MN) following the manufacturer’s instructions.

### Immunofluorescence

Briefly, cells seeded on coverslips were fixed with 4% (w/v) paraformaldehyde (Sigma) for 10 min and permeabilized with 0.1% (v/v) Triton X-100 for 5 min at room temperature. The cells were then incubated overnight with primary antibodies at 4°C, followed by incubation with fluorescent secondary antibody (invitrogen) for 1 hour at room temperature. After final washes with PBS, the coverslips were mounted using an anti-fade mounting solution containing 4′,6-diamidino-2-phenylindole (DAPI; Vector lab, Burlingame, CA) and images were examined and captured.

### Cell invasion assay

Invasion was measured by using 24-well BioCoat cell culture inserts (BD Biosciences, NJ) with an 8-μm–porosity polyethylene terephthalate membrane coated with Matrigel Basement Membrane Matrix.

### Nude mice orthotopic model study

The study was approved by the committee on the use of live animals in teaching and research of the Harbin Medical University, Harbin, China. Experiments were started after 1 week of acclimatization. For assessment of LBH589 inhibits the proliferation of HCC-LM3 tumors in orthotopic tumor xenografts, an orthotopic liver tumor model in nude mice was established. Briefly, we used HCC-LM3, HCC-LM3 (which transfected with gankyrin) and HCC-LM3 (which transfected with vector ctrl) cells. Then these approximately 1 × 10^7^ HCC-LM3 cells in 0.2 ml culture medium phosphate buffered saline (PBS) were injected subcutaneously into the right flank of the mice, which were then observed daily for signs of tumor development. Once the subcutaneous tumor reached 1–1.5 cm in diameter, it was removed and cut into about 1–2 mm^3^ cubes which were implanted into the left liver lobe of another group of nude mice. Animals were randomized to receive either LBH589 (10 mg/kg/d, i.p.) or vehicle (saline solution with 5% DMSO and 1% Tween 80) at 1 week after implantation. Liver tumors were harvested for experiment at 5 weeks after tumor implantation. Tumor volume was calculated as below: V (cm^3^) = width^2^ (cm^2^) × length (cm) /2.

### In vivo metastasis analysis

HepG2, HepG2 (which transfected with gankyrin) and HepG2 (which transfected with vector ctrl) cells (1 × 10^6^/0.2 ml) were injected into nude mice by way of tail vein to imitate tumor metastasis. Experimental animals (n = 8/group) received either LBH589 (10 mg/kg/d, i.p.) or vehicle (saline solution with 5% DMSO and 1% Tween 80) five times per week beginning on the day of implantation. The mice were killed 5 weeks after the inoculation and lungs were removed and fixed in formaldehyde. The lung metastases were confirmed by H&E staining.

### Immunohistochemistry analysis

Immunohistochemistry was performed as described previously [10] using Ki-67, cleaved-caspase-3, CD31, E-cadherin, N-cadherin and vimentin antibodies. In brief, tissue sections were deparaffinized in xylene and rehydrated with ethanol. Tissue sections were then preincubated with 10% normal goat serum in PBS (pH 7.5) followed with incubation with primary antibody overnight at 4°C. Tissue sections were then stained with biotinylated secondary antibody (Vector lab) for 1 hour at room temperature, followed by the Vectastain Elite ABC reagent (Vector lab) for 30 min. The peroxidase reaction was developed with diaminobenzidine (DAB kit; Vector lab) and the slides were counterstained with hematoxylin.

### Statistical analysis

All the data are expressed as mean values ± standard deviation (SD). Comparisons among multiple groups were made with a one-way analysis of variance (ANOVA) followed by Dunnet t-test. “*p* <0.05′ was used for statistical significance.

## Results

### LBH589 is a potent anti-HCC agent and induces histone acetylation and apoptosis in HCC cells

**Figure 1:**
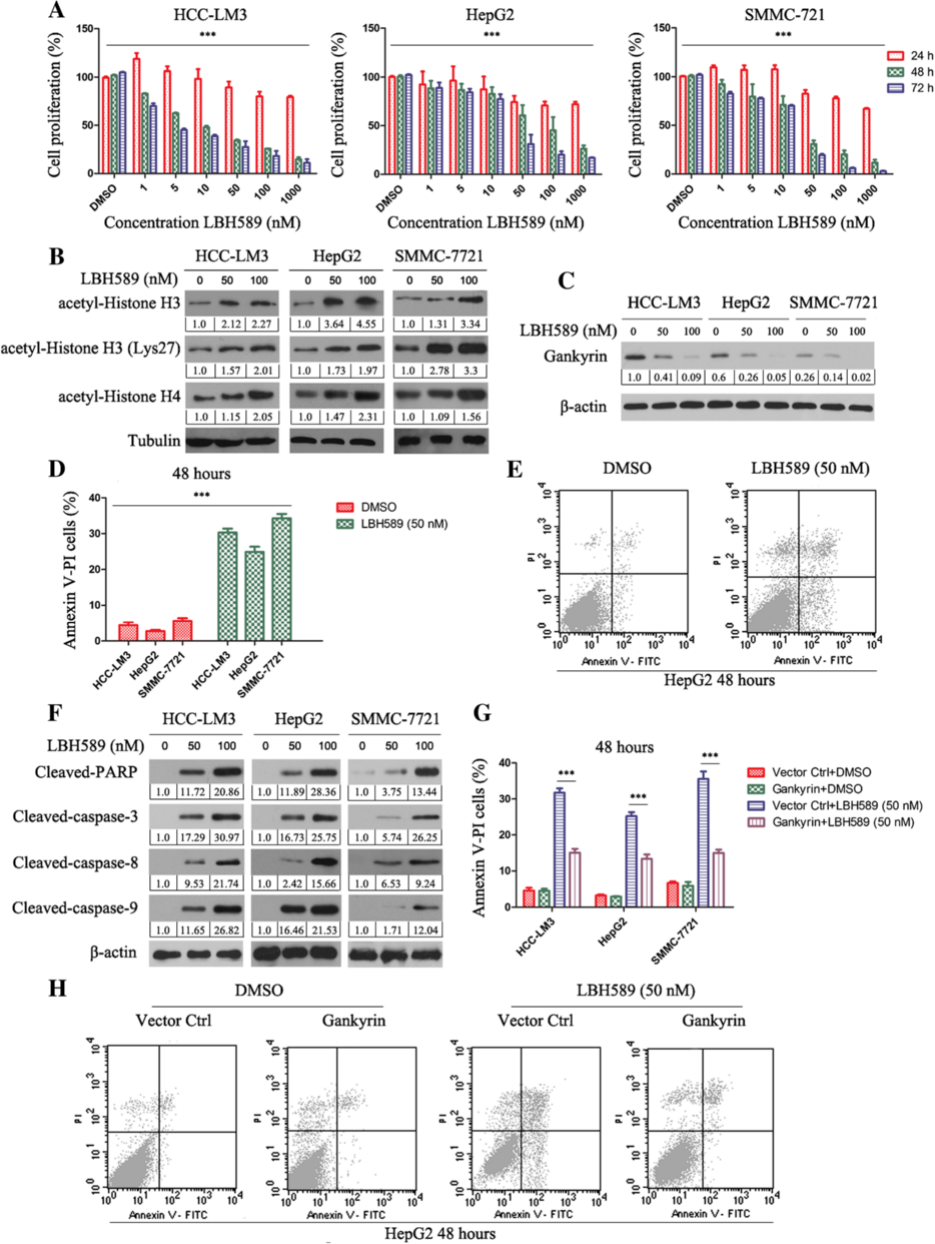
**LBH589 is cytotoxic to HCC cells in a dose- and time-dependent manner. (A)** MTT assay showing percentage of viable HCC cells treated with DMSO, 1, 5, 10, 50, 100 and 1000 nM of LBH589 for 24, 48 and 72 h. The results represent means ± SD of experiments performed in triplicate. ***P < 0.001, LBH589 treated versus control group. **(B)** Expression of acetyl-Histone H3, acetyl-Histone H3 (Lys27) and acetyl-Histone H4 were determined via western blotting after treatment with 50 and 100 nmol/L LBH589 for 24 h. Tubulin was used as the internal control. All assays were done in triplicate. **(C)** The expression of gankyrin after treatment with 50 and 100 nmol/L LBH589 for 24 h. β-actin was used as the internal control. All assays were done in triplicate. **(D)** Flow cytometry results of annexin V-PI staining of HCC cells after exposure to DMSO or LBH589 (50 nM) for 48 h. The results represent means ± SD of experiments performed in triplicate. ***P < 0.001, LBH589 treated versus DMSO group. **(E)** A representative example of apoptosis of HepG2 cell line treated with 50 nM of LBH589 at 48 h is shown. **(F)** Expression of apoptosis relative proteins were determined via western blotting after treatment with 50 and 100 nmol/L LBH589 for 24 h. β-actin was used as the internal control. All assays were done in triplicate. **(G)** Flow cytometry results showed gankyrin overexpression attenuated the LBH589-induced apoptosis of HCC cells. The results represent means ± SD of experiments performed in triplicate. ***P < 0.001, LBH589 + vector ctrl treated versus LBH589 + gankyrin group. **(H)** A representative example of apoptosis of HepG2 cell line (which transfected human gankyrin/control plasmid) treated with 50 nM of LBH589 at 48 h.

Exposure of HCC-LM3, HepG2 and SMMC-7721 cells to LBH589 for 24, 48 and 72 hours resulted in a significant growth inhibition (Figure 1A). To verify whether LBH589 induces hyperacetylation of histones in HCC cells with different concentrations of LBH589 (50 and 100 nM) for 24 h, acetylation of histone H3, histone H3 (Lys27) and histone H4 were analyzed by western blotting. Results suggest that HCC cells exhibited a progressive increase in histone H3, histone H3 (Lys27) and histone H4 acetylation correlating with LBH589 dose of treatment (Figure 1B).

To determine whether HCC cell death induced by LBH589 involves apoptosis, flow cytometric analysis with annexin V–PI staining was performed. LBH589 induced apoptosis in all HCC cell lines tested in the dose of 50 nM (Figure 1D). Figure 1E is a representative example of apoptosis of HepG2 cell line treated with 50 nM of LBH589 at 48 h.

### LBH589 decreases gankyrin and induces cell death in a caspase-dependent manner by cleavage of caspases 3, 8 and 9

Next we explored the effect of LBH589 on apoptotic pathways. LBH589 significantly decreased the expression of gankyrin (Figure 1C), and induced cleavage of caspases 3, 8 and 9, as well as PARP, in a dose-dependent manner after 24-hr incubation with the drug (Figure 1F). Figure 1C showed the basal level of gankyrin in HCC-LM3 and HepG2 was stronger than SMMC-7721. Additional file 1: Figure S1A showed the invasive capability of HCC-LM3 and HepG2 was stronger than SMMC-7721 cell. In order to determine the significance of gankyrin, we transfected human gankyrin plasmid into HCC cells. Additional file 1: Figure S1B showed the expression of myc after selection with G418. Gankyrin overexpression attenuated the LBH589-induced apoptosis of HCC cells (Figure 1G). Figure 1H is a representative example of apoptosis of HepG2 cell line (which transfected human gankyrin/control plasmid) treated with 50 nM of LBH589 at 48 h. Transient transfection of pCMV-HA-gankyrin also can attenuate the LBH589-induced apoptosis of HCC cells (Additional file 2: Figure S2B). Additional file 2: Figure S2A showed the expression of HA after transient transfection of pCMV-HA-gankyrin.

### LBH589 decreases the levels of p-STAT3 and p-Akt in HCC cells, and gankyrin overexpression can attenuate the effect of LBH589

**Figure 2:**
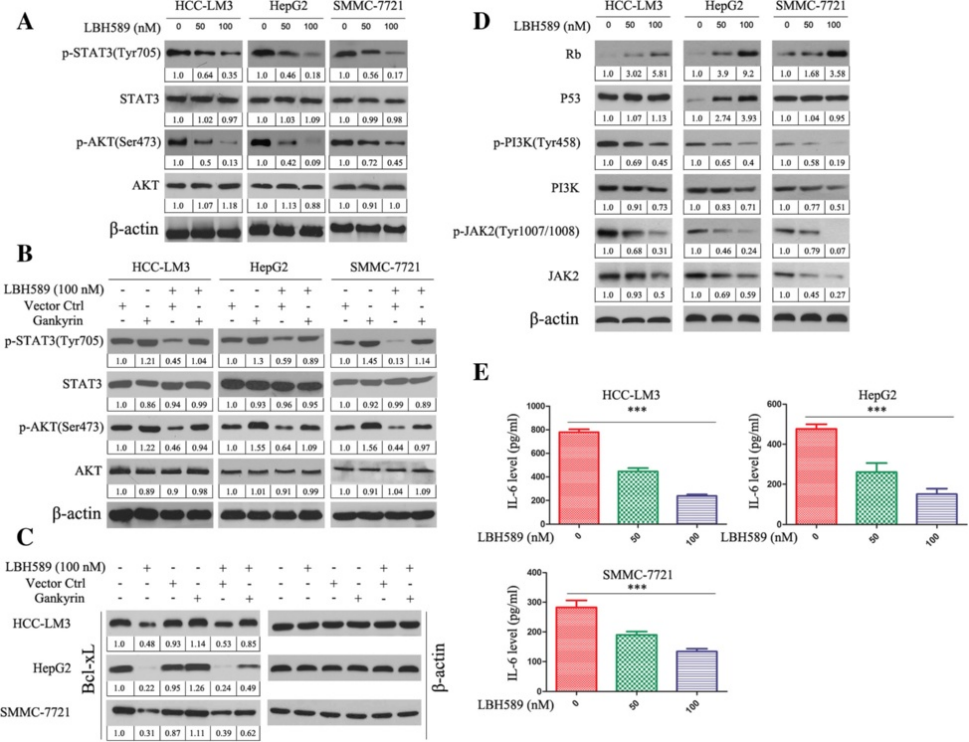
**LBH589 decreases the levels of p-STAT3, p-Akt and Bcl-xL in HCC cells, and gankyrin overexpression can attenuate the effect of LBH589.** LBH589 mediates the expression of p-Akt and p-STAT3 through gankyrin/PI3K/Akt and gankyrin/Rb/IL-6/JAK2/STAT3 pathways. **(A)** HCC cells were treated for 24 h with or without LBH589 and analyzed for the indicated protein by western blotting. **(B)** Western blotting results showed LBH589 induced Akt and STAT3 dephosphorylation was reduced by gankyrin overexpression. **(C)** Western blotting results showed LBH589 decreased Bcl-xL expression and overexpression of gankyrin partially protected against LBH589-induced inhibition of Bcl-xL. β-actin was used as the internal control. All assays were done in triplicate. **(D)** LBH589 increased Rb expression and decreased the expression of PI3K, p-PI3K (Tyr458), JAK2 and p-JAK2 (Tyr1007/1008) in three HCC cells. After treatment of LBH589, the expression of p53 increased in HepG2, no obvious change was detected in HCC-LM3 and SMMC-7721 cells. β-actin was used as the internal control. All assays were done in triplicate. **(E)** After treatment of LBH589, the level of IL-6 in three HCC cells was significantly decreased compared with controls. The results represent means ± SD of experiments performed in triplicate. ***P < 0.001, LBH589 treated versus control group.

We first evaluated the effect of LBH589 on the expression of p-STAT3 and p-Akt in HCC cells. Figure 2A shows that treatment of HCC cells with LBH589 for 24 h leads to a significant reduction in serine-phosphorylated Akt expression as well as tyrosine-phosphorylated STAT3 although total Akt and STAT3 were unaffected. Next, we examined whether gankyrin overexpression could inhibit LBH589-induced dephosphorylation of Akt and STAT3 in HCC cell lines. As shown in Figure 2B, gankyrin overexpression activated the expression of p-Akt and p-STAT3, and LBH589 induced Akt and STAT3 dephosphorylation was reduced by gankyrin overexpression. Gankyrin knockdown also can decrease the expression of p-Akt and p-STAT3 (Additional file 3: Figure S3B). Additional file 3: Figure S3A showed the expression of gankyrin after transfection of Lenti-shgankyrin. The results indicate that gankyrin/STAT3/Akt pathway is likely an important target of LBH589 in HCC cells.

### LBH589 downregulates Bcl-xL expression, and overexpression of gankyrin partially protects against LBH589-mediated inhibition of Bcl-xL

Next, we investigated Bcl-xL, one of the key regulators of apoptosis in HCC cells is considered important for HCC cell survival and drug resistance [11]. As shown in Figure 2C, LBH589 treatment strongly downregulated Bcl-xL expression in HCC cells. Furthermore, overexpression of gankyrin using human gankyrin plasmid partially protected against LBH589-induced inhibition of Bcl-xL (Figure 2C), indicating that reduction in Bcl-xL may contribute an important role in LBH589-induced apoptosis in HCC cells.

### LBH589 mediates p-Akt and p-STAT3 expression through gankyrin/PI3K/Akt and gankyrin/Rb/IL-6/JAK2 pathways

Next, we investigated the expression of p53 and Rb, which are the direct targets of gankyrin. After treatment of LBH589, the expression of p53 increased in HepG2, no obvious change was detected in HCC-LM3 and SMMC-7721 cells. After LBH589 treatment, the expression of Rb increased in three HCC cells (Figure 2D). To further elucidate how LBH589 mediate p-Akt and p-STAT3 through gankyrin. We detected the effect of LBH589 on P13K and JAK2 expression. The expression of p-PI3K (Tyr458) and PI3K decreased after LBH589 treatment in three HCC cells (Figure 2D), which results in inhibition of p-Akt activity (Figure 2A). This result suggests a mechanism in which LBH589 inhibits p-Akt signaling through control of the gankyrin/PI3K/Akt pathway.

Santhanam *et al.* and Zhu *et al.* reported that Rb can decrease the interleukin-6 (IL-6) level [12,13]. and IL-6 can increase the expression of p-STAT3. LBH589 increased the expression of Rb in three HCC cells, and then we detected the levels of IL-6 in supernatant decreased in three HCC cells (Figure 2E). Western blotting showed the expression of p-JAK2 (Tyr1007/1008) and JAK2 decreased after LBH589 treatment (Figure 2D). And gankyrin knockdown also can decreased the levels of IL-6 (Additional file 3: Figure S3C). So the results suggest LBH589 inhibits p-STAT3 through gankyrin/Rb/IL-6/JAK2 pathway.

### LBH589 inhibits invasive potential of HCC cells in vitro

**Figure 3:**
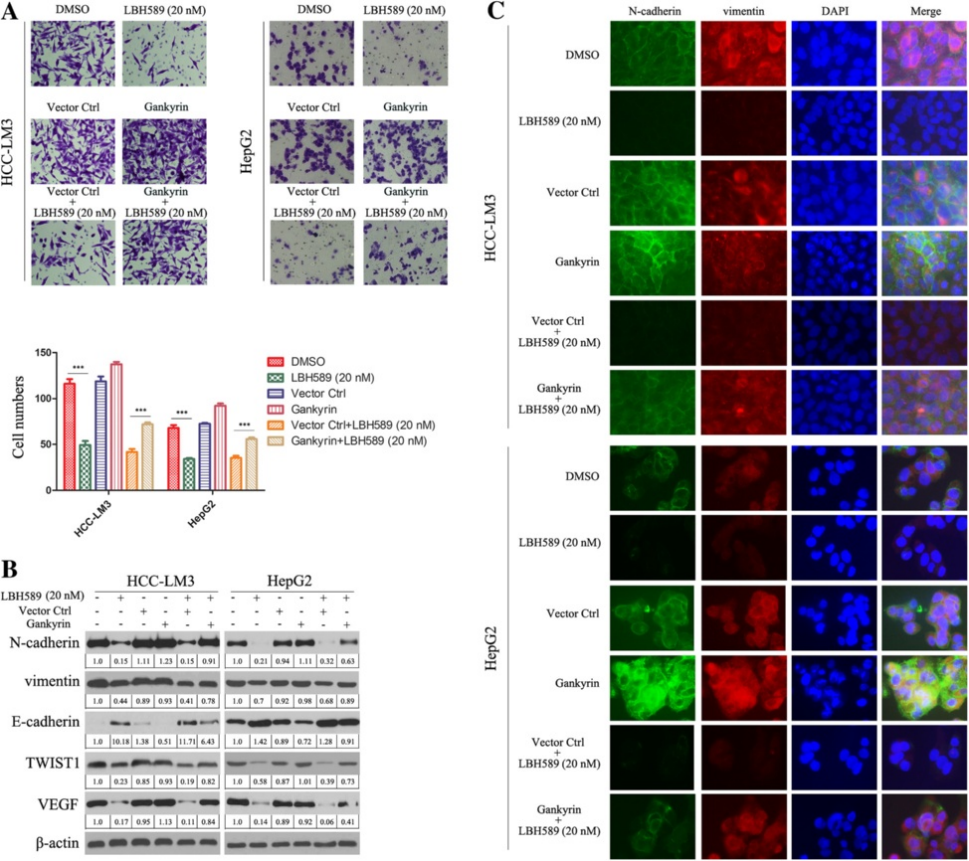
**LBH589 inhibits invasive potential of HCC cells *in vitro*. (A)** Cell invasion experiment results showed LBH589 significantly inhibited the invasive capacity of HCC-LM3 and HepG2 cells, and gankyrin overexpression attenuated the LBH589-induced inhibition of invasion in HCC-LM3 and HepG2 cells. ***P < 0.001. The results represent means ± SD of experiments performed in triplicate. **(B)** Western blotting showed LBH589 increased the expression of E-cadherin and decreased the expression of N-cadherin, vimentin, VEGF and TWIST1 in HCC-LM3 and HepG2 cells. Overexpression of gankyrin abrogated the effect of LBH589-induced reduction of EMT and angiogenesis. β-actin was used as the internal control. All assays were done in triplicate. **(C)** Single and merged images were taken to show immunofluorescence staining of N-cadherin (green) and vimentin (red) accompanied by the cell nucleus (blue) stained by DAPI.

To determine the function of LBH589, we treated HCC-LM3 and HepG2 with LBH589. LBH589 significantly inhibited their invasive capacity by 2.9- and 2.5-fold, as compared with DMSO-treated cells (Figure 3A). In contrast, gankyrin overexpression in HCC-LM3 and HepG2 cells attenuated the LBH589-induced inhibition of invasion (Figure 3A). Transient transfection of pCMV-HA-gankyrin also can attenuate the LBH589-induced inhibition of invasion (Additional file 2: Figure S2C). Given that LBH589 inhibits HCC invasion, we investigated the effect of LBH589 on epithelial-mesenchymal transition (EMT), a critical event in tumor invasion. Western blotting detected higher expression of E-cadherin in HCC-LM3 and HepG2 cells treated with LBH589. In contrast, the expression of N-cadherin, vimentin, VEGF and TWIST1 decreased in LBH589 treated HCC-LM3 and HepG2 cells (Figure 3B). Overexpression of gankyrin abrogated the effect of LBH589-induced reduction of EMT (Figure 3B). As shown by immunofluorescence (Figure 3C), LBH589 markedly reduced N-cadherin and vimentin levels in both HCC-LM3 and HepG2 cells. Overexpression of gankyrin abrogated the effect of LBH589-induced reduction of N-cadherin and vimentin, which was in conjunction with the results in Figure 3B. The immunofluorescence results for E-cadherin are shown in Additional file 4: Figure S4, LBH589 markedly increased E-cadherin level in both HCC-LM3 and HepG2 cells. Overexpression of gankyrin abrogated the effect of LBH589-induced induction of E-cadherin.

### LBH589 increases p16 and p27 expression, downregulates cyclin D1 and induces G1 cell cycle arrest in HCC cells

**Figure 4:**
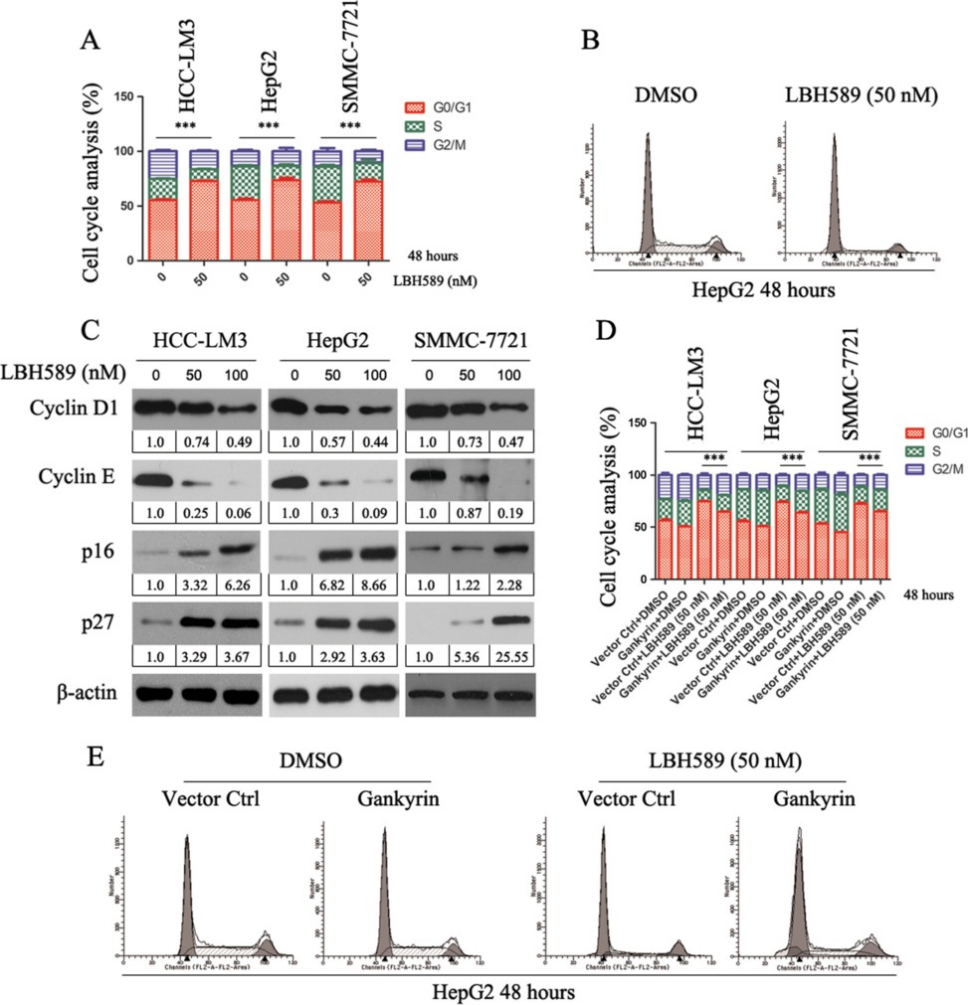
**Effect of LBH589 on cell cycle proteins and cell cycle progression. (A)** Cell cycle analysis in LBH589 treated HCC cells showing arrest in G1 phase. The results represent means ± SD of experiments performed in triplicate. ***P < 0.001, LBH589 treated versus control group. **(B)** A representative example of cell cycle arrest of HepG2 cell line treated with 50 nM of LBH589 at 48 h. **(C)** LBH589 induces expression of p16 and p27 and reduces expression of cyclin D1 and cyclin E. β-actin was used as the internal control. All assays were done in triplicate. **(D)** Gankyrin overexpression attenuated the LBH589-induced G0/G1 phase arrest of HCC cells. ***P < 0.001, the results represent means ± SD of experiments performed in triplicate. **(E)** A representative example of cell cycle arrest of HepG2 cell line (which transfected human gankyrin/control plasmid) treated with 50 nM of LBH589 at 48 h.

To further investigate the effect of LBH589 on cell cycle distribution in HCC cells, HCC cells were incubated with 50 nM LBH589 for 48 h. The FACs analysis revealed a more distinguished decrease in the number of cells in S phase at 48 h compared with DMSO group (Figure 4A). The data here suggested that the cell cycle was blocked at G0/G1 checkpoint more significantly. Figure 4B is a representative example of cell cycle arrest of HepG2 cell line treated with 50 nM of LBH589 at 48 h.

We investigated the effect of LBH589 on their expression as the cell cycle promoter cyclin D1 and cyclin E are key regulators of G1 phase. Shown in Figures 4C, we observed a reduction in cyclin D1 and E after treated with LBH589 for 24 h. As increased expression of p27 results in inhibition of proliferation, we examined the effect of LBH589 on p27 expression and that of p16, another cell cycle inhibitor that has been shown to be transcriptionally silenced in HCC [14]. Expression of both p27 and p16 proteins was induced by LBH589 after 24 h (Figure 4C).

In order to determine the significance of gankyrin, we transfected human gankyrin plasmid into HCC cells. Gankyrin overexpression attenuated the LBH589-induced G0/G1 phase arrest of HCC cells (Figure 4D). Figure 4E is a representative example of cell cycle arrest of HepG2 cell line (which transfected human gankyrin/control plasmid) treated with 50 nM of LBH589 at 48 h. Transient transfection of pCMV-HA-gankyrin also can attenuate the LBH589-induced G0/G1 phase arrest of HCC cells (Additional file 2: Figure S2D).

### LBH589 inhibits localized growth and metastasis of HCC in vivo

**Figure 5:**
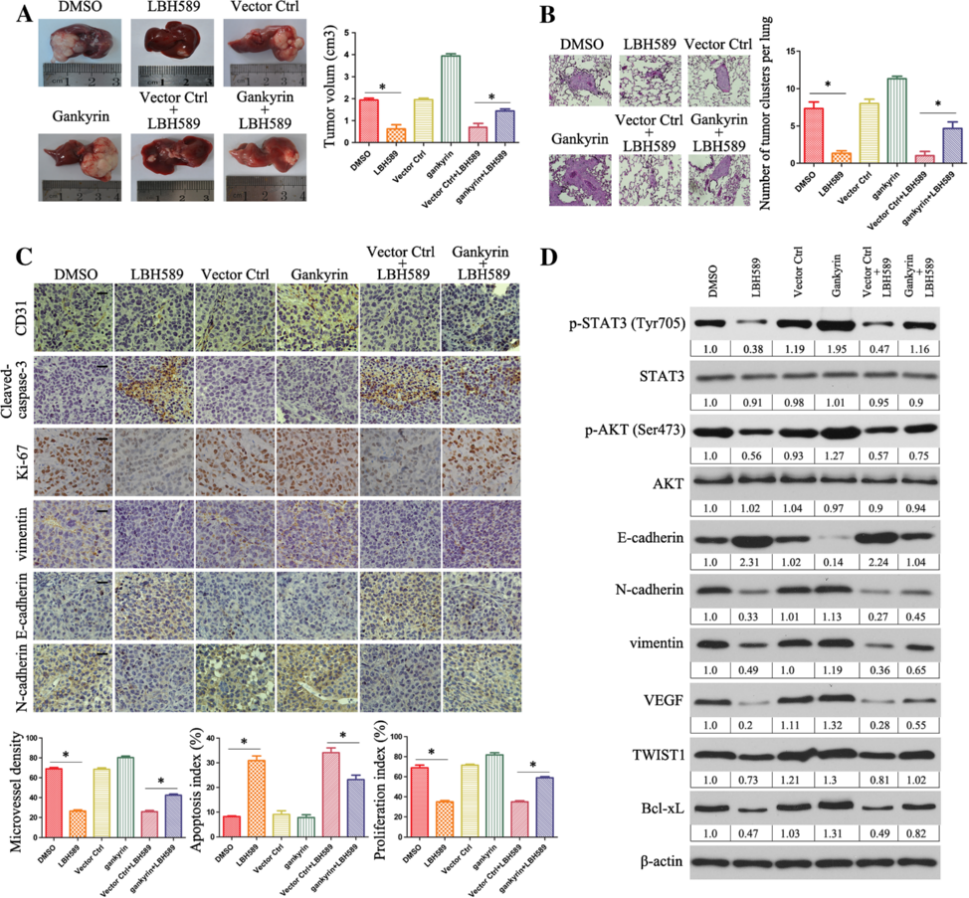
**LBH589 inhibits proliferation and metastasis of HCC *in vivo*. (A)** Photomicrographs were taken for liver tumors in nude mice, 5 weeks after orthotopic xenograft transplantation of HCC-LM3, vector ctrl transfected or gankyrin transfected HCC-LM3 cells. Representative images of a mouse in each group were presented. Tumor volumes from each group were measured as described in Materials and Methods. **(B)** Representative lung tissue sections from each group were shown (hematoxylin and eosin stain; magnification, × 100). The number of lung metastatic foci in each group was calculated. **(C)** Tumors from different groups were immunostained for indicated molecules. CD31-stained microvessels were counted to record microvessel density; Apoptosis cells were counted to give the apoptosis index; and cells expressing Ki-67 were counted to calculate the proliferation index. Pictures are representative of three independent experiments. Black scale bars, 100 μm. **(D)** Western blotting was performed to detect expression of indicated molecules in tumor samples. β-actin was used as the internal control. All assays were done in triplicate. *P < 0.05, the results expressed as means ± SD of three independent experiments.

We further examined the effect of LBH589 on HCC growth by establishing an orthotopic liver tumor model in nude mice, and examined the effect of LBH589 on pulmonary metastasis by injecting HCC cells through tail vein to imitate tumor metastasis. HCC-LM3 and HepG2 cells were used for *in vivo* studies. Compared to DMSO groups, LBH589 treatment resulted in significant decrease of tumor size, the number of pulmonary metastatic foci and average size of pulmonary metastatic lesions (Figure 5A-B). Furthermore, the orthotopic liver tumor model and pulmonary metastasis model based on HCC-LM3 and HepG2 cells also showed that gankyrin overexpression attenuated the effect of LBH589-induced reduction of tumor cell proliferation and lung metastasis (Figure 5A-B). The IHC analysis showed the changes of Ki-67, cleaved-caspase-3, CD31, E-cadherin, N-cadherin and vimentin in different group (Figure 5C). The expression of relative proteins mentioned above were also analyzed by western blotting in different group (Figure 5D). Together, these results reveal functional significance of LBH589 with high propensity to inhibit proliferation and metastasis in HCC and in aggressive tumors.

## Discussion

HCC is one of the most difficult cancer to treat, largely due to the advanced stage by the time it is diagnosed and poor response to treatment, and its incidence is rising in industrialized nations. Improvement in both chemoprevention and treatment of HCC is sorely needed. As a new class of chemotherapeutic agents, HDACi have demonstrated potent anticancer activities in preclinical studies and are currently in various stages of clinical development. LBH589 (Novartis Pharma AG) is a hydroxamic acid derivative, which has been reported to possess cytotoxic properties against different human cancers *in vivo* and *in vitro*[15-17]. But the exact final pathways that lead to the anticancer effects observed still remain to be fully elucidated.

**Figure 6:**
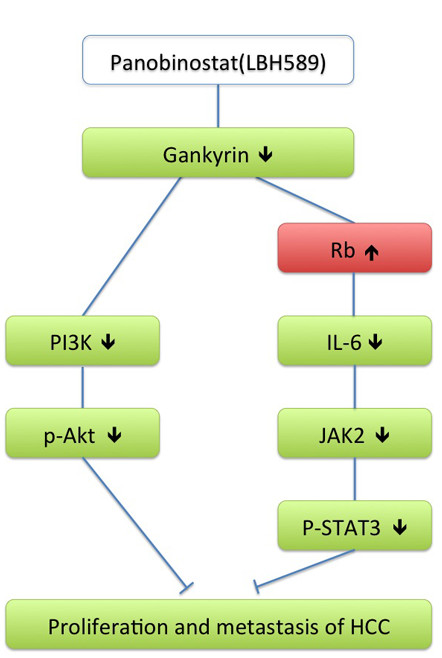
The proposed mechanisms by which LBH589 exerts its effects.

Given the fact that liver is well protected HCC by the tumor suppressor proteins p53, Rb and C/EBPα, it could be assumed that the development of HCC might include activation of a powerful system for the elimination of these proteins. During the examination of early events in hepatocarcinogenesis both in animal models and human HCC, gankyrin has been identified as a candidate for this important role [18]. The studies of gankyrin-dependent promotion of liver cancer have indicated that gankyrin could not only bind to mdm2 and enhance degradation of p53 but also interact with Rb to reduce its stability. Gankyrin could also bind to CDK4 and replaces p16 from CDK4, leading to the activation of CDK4 [19,20]. Against the significant role of each of these proteins in protection of liver from HCC, one could assume the elevation of gankyrin might be a key step in the release of growth inhibitory control of the liver and in development of liver cancer. We, therefore, investigated the effects of a novel HDACi, LBH589, in HCC cell lines. We show that LBH589 has a significant inhibitory effect on gankyrin/STAT3/Akt signaling and EMT, downregulating the expression of gankyrin and blocking phosphorylation of STAT3 and Akt, thereby inducing inhibition of proliferation and metastasis. We presume that LBH589 mediates the expression of gankyrin from transcriptional level (e.g. we presume that LBH589 enhances the activity of farnesoid X receptor (FXR), through which the expression of gankyrin can be inhibited at transcriptional level [21]) either from translational level (e.g. we presume that LBH589 inhibits the expression of hepatoma upregulated protein (HURP), HURP inhibition can activate the MDM2-mediated ubiquitination and degradation of gankyrin [22].), or from both transcriptional and translational level simultaneously. But the exact mechanisms should be explored through further study in the future. To explore how LBH589 blocks phosphorylation of STAT3 and Akt, we performed western blotting to detect the expression of PI3K, Rb and JAK2 after LBH589 treatment. And we detected the levels of IL-6 in supernatant in three HCC cells after LBH589 treatment. The results showed LBH589 inhibits the expression of p-Akt through gankyrin/PI3K/Akt pathway. And LBH589 inhibits p-STAT3 through gankyrin/Rb/IL-6/JAK2/STAT3 pathway. After treatment of LBH589, the expression of p53 increased in HepG2, no obvious change was detected in HCC-LM3 and SMMC-7721 cells. So we think LBH589 inhibits the proliferation and metastasis of HCC is p53 independent. The potential mechanisms of LBH589 are summarized in Figure 6.

LBH589 also decreases the expression of cyclin D1, cyclin E, Bcl-xL, N-cadherin, vimentin, VEGF and TWIST1, the major downstream targets of STAT3 and Akt. LBH589 upregulates the expression of p27 and p16, then inhibits cell cycle progression. And overexpression of gankyrin partly protects against LBH589-induced HCC cell death and metastatic inhibition. Finally, LBH589 inhibits localized growth and metastasis of HCC *in vivo*.

Our MTT assay demonstrated that the LBH589 could induce a dramatic cell viability reduction in all the three HCC cell lines tested. After cells were treated with LBH589 for 48 hours, we observed a significant decrease of S-phase population. FACS analysis also showed that the growth inhibitory effect by LBH589 was also related to induction of apoptosis in HCC cells. Using western blotting assay, we found that gankyrin was decreased significantly after LBH589 treatment. To evaluate the essentiality of gankyrin in LBH589 mediated growth inhibition, human gankyrin plasmid was transfected into HCC cells. After transfection, we found HCC cells with high-level gankyrin can significantly attenuate the cell growth inhibitory effect of LBH589. Therefore, we propose that gankyrin might contribute, at least partially, to LBH589 induced tumor growth inhibition.

The mechanisms of HDACi-induced cytotoxicity may vary depending on the class of HDAC being inhibited and the downstream targets of HDAC in different cancer cells. Our results in HCC show that LBH589-induced apoptosis is associated with cleavage of caspases 3, 8 and 9, and PARP cleavage. Further, LBH589-induced apoptosis is in large part dependent on caspase activation. In HCC cells, LBH589 also modulates the expression of the antiapoptotic proteins. The expression of Bcl-xL was significantly reduced, and overexpression of gankyrin can attenuate the LBH589-induced inhibition of Bcl-xL.

We further demonstrate that incubation of HCC cells with LBH589 leads to the loss of N-cadherin and vimentin and accumulation of E-cadherin, and LBH589 significantly inhibited the invasive capacity of HCC cells. Conversely, gankyrin overexpression attenuates LBH589-induced metastatic inhibition. We think that these results might apply to a number of additional cancer types other than HCC because gankyrin is frequently upregulated in many other cancer types as well [23-26].

The effect of LBH589 on HCC proliferation, invasion and metastasis was also directly demonstrated in our *in vivo* studies. In orthotopic xenografts and *in vivo* metastasis analysis, LBH589 group generated smaller primary tumors and fewer lung metastasis foci, indicating LBH589 inhibited aggressive and metastatic properties of HCC. Moreover, up-regulation of gankyrin led to severe inhibition of LBH589-induced suppression of tumor growth and lung metastasis of HCC in mice. To our knowledge, this is the first report that gankyrin is critical for LBH589 to inhibit HCC metastasis, in addition to tumor suppression, proliferation and growth.

## Conclusions

In conclusion, we have demonstrated for the first time that LBH589 could inhibit expression of gankyrin and metastasis in different HCC cell lines. LBH589 induced cell cycle arrest and apoptosis *in vitro* and inhibited tumor growth and metastasis in a nude mice model. Its ability to target mainly the gankyrin/STAT3/Akt cellular pathway suggests its viability as part of the therapeutic armamentarium for HCC. Our results provide preclinical rationale for clinical development of LBH589 for HCC.

## Abbreviations

HCC: Hepatocellular carcinoma; HDAC: Histone deacetylase; EMT: Epithelial-mesenchymal transition.

## Competing interests

The authors declare that they have no competing interests.

## Authors’ contributions

XS, JW and TZ contributed equally to this work. XS, JW and TZ designed and carried out experimental works. RS performed the data collection. YL, DY and SP participated in the research. NB and JL participated in discussion. HJ and LL supervised the project, analyzed data and wrote the paper. All authors read and approved the final manuscript.

## Supplementary Material

Additional file 1: Figure S1 The invasive capability of three HCC cells *in vitro*. (A) Cell invasion assay was performed in the indicated cells. Data are presented as mean ± SD from three independent experiments. ***P < 0.001. (B) The levels of myc after selection with G418. β-actin was used as the internal control. All assays were done in triplicate. Click here for file

Additional file 2: Figure S2 Transient transfection of pCMV-HA-gankyrin can attenuate the effect of LBH589. (A) The expression of HA after transient transfection of pCMV-HA-gankyrin in three HCC cells. β-actin was used as the internal control. All assays were done in triplicate. (B) Flow cytometry results showed transient transfection of gankyrin attenuated the LBH589-induced apoptosis of HCC cells. The results represent means ± SD of experiments performed in triplicate. ***P < 0.001, LBH589 + vector ctrl treated versus LBH589 + HA-gankyrin group. (C) Cell invasion experiment results showed transient transfection of gankyrin attenuated the LBH589-induced inhibition of invasion in HCC-LM3 and HepG2 cells. ***P < 0.001. The results represent means ± SD of experiments performed in triplicate. (D) Transient transfection of Gankyrin attenuated the LBH589-induced G0/G1 phase arrest of HCC cells. ***P < 0.001, the results represent means ± SD of experiments performed in triplicate. Click here for file

Additional file 3: Figure S3 Gankyrin knockdown can decrease the levels of p-Akt, p-STAT3 and IL-6 in HCC cells. (A) The expression of gankyrin after transfection of Lenti-shgankyrin. β-actin was used as the internal control. All assays were done in triplicate. (B) The expression of p-Akt and p-STAT3 decreased after transfection of Lenti-shgankyrin. β-actin was used as the internal control. All assays were done in triplicate. (C) After transfection of Lenti-shgankyrin, the level of IL-6 in three HCC cells was significantly decreased compared with controls. The results represent means ± SD of experiments performed in triplicate. ***P < 0.001, Lenti-shgankyrin group versus control group. Click here for file

Additional file 4: Figure S4 Single and merged images were taken to show immunofluorescence staining of E-cadherin (green) accompanied by the cell nucleus (blue) stained by DAPI in HCC-LM3 and HepG2 cells. Click here for file
