# Supplementary figures and images for: LBH589 Inhibits proliferation and metastasis of hepatocellular carcinoma via inhibition of gankyrin/stat3/akt pathway
Source: Mol Cancer. 2013 Oct 5;12:114. doi: 10.1186/1476-4598-12-114 (PMC3853770; doi:10.1186/1476-4598-12-114)

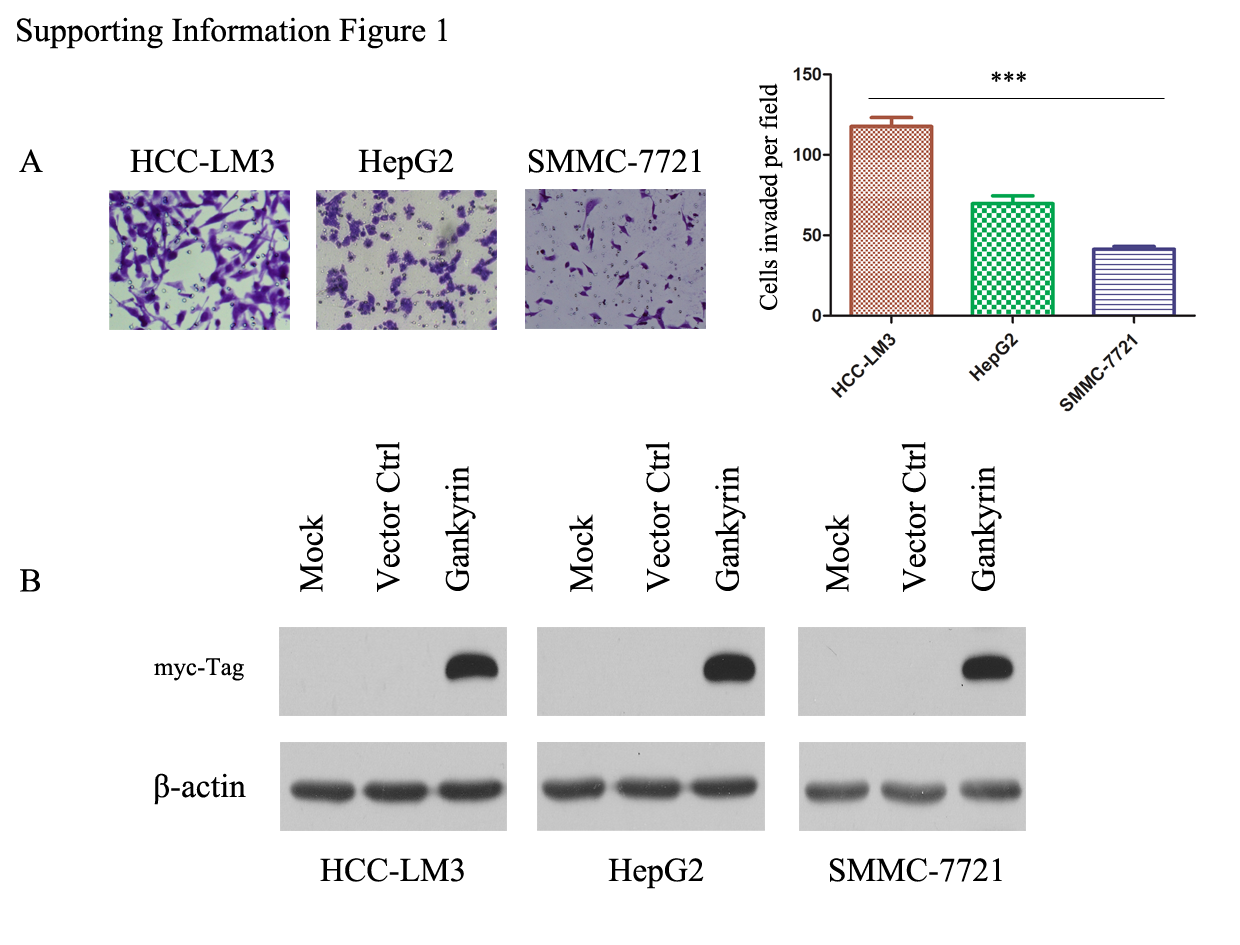

Supplement: Additional file 1: Figure S1 — The invasive capability of three HCC cells in vitro. (A) Cell invasion assay was performed in the indicated cells. Data are presented as mean ± SD from three independent experiments. ***P < 0.001. (B) The levels of myc after selection with G418. β-actin was used as the internal control. All assays were done in triplicate. [file 1476-4598-12-114-S1.tiff]

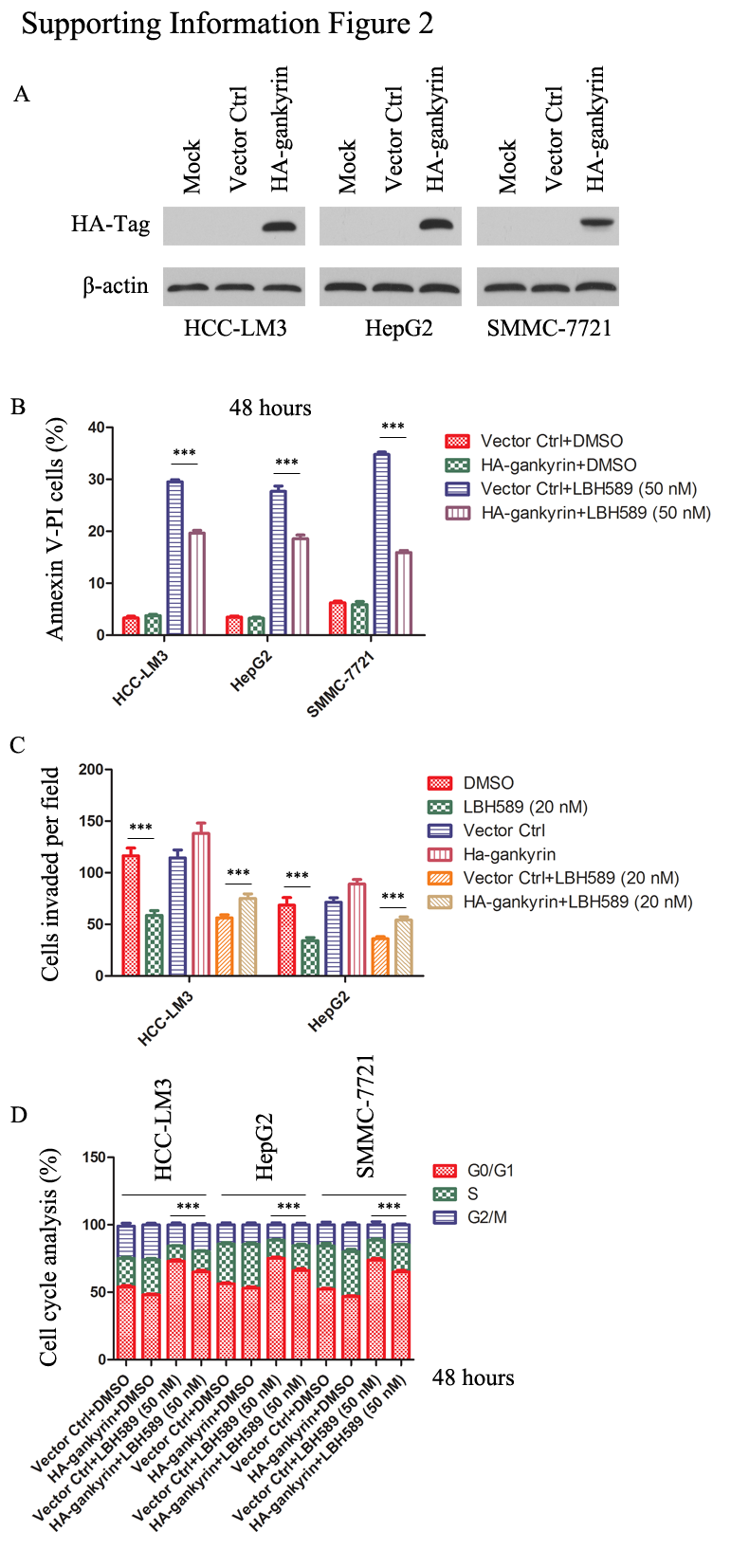

Supplement: Additional file 2: Figure S2 — Transient transfection of pCMV-HA-gankyrin can attenuate the effect of LBH589. (A) The expression of HA after transient transfection of pCMV-HA-gankyrin in three HCC cells. β-actin was used as the internal control. All assays were done in triplicate. (B) Flow cytometry results showed transient transfection of gankyrin attenuated the LBH589-induced apoptosis of HCC cells. The results represent means ± SD of experiments performed in triplicate. ***P < 0.001, LBH589 + vector ctrl treated versus LBH589 + HA-gankyrin group. (C) Cell invasion experiment results showed transient transfection of gankyrin attenuated the LBH589-induced inhibition of invasion in HCC-LM3 and HepG2 cells. ***P < 0.001. The results represent means ± SD of experiments performed in triplicate. (D) Transient transfection of Gankyrin attenuated the LBH589-induced G0/G1 phase arrest of HCC cells. ***P < 0.001, the results represent means ± SD of experiments performed in triplicate. [file 1476-4598-12-114-S2.tiff]

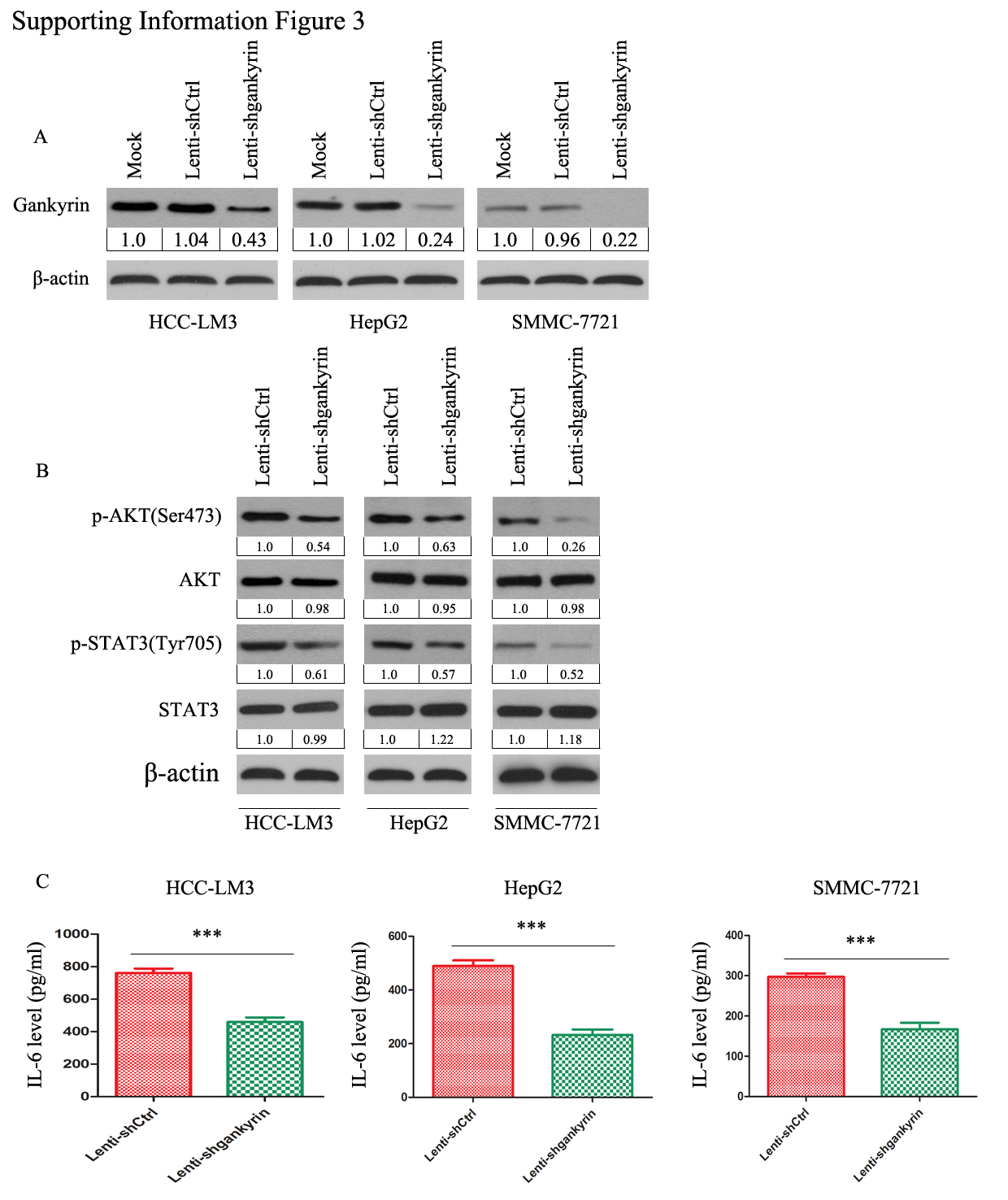

Supplement: Additional file 3: Figure S3 — Gankyrin knockdown can decrease the levels of p-Akt, p-STAT3 and IL-6 in HCC cells. (A) The expression of gankyrin after transfection of Lenti-shgankyrin. β-actin was used as the internal control. All assays were done in triplicate. (B) The expression of p-Akt and p-STAT3 decreased after transfection of Lenti-shgankyrin. β-actin was used as the internal control. All assays were done in triplicate. (C) After transfection of Lenti-shgankyrin, the level of IL-6 in three HCC cells was significantly decreased compared with controls. The results represent means ± SD of experiments performed in triplicate. ***P < 0.001, Lenti-shgankyrin group versus control group. [file 1476-4598-12-114-S3.tiff]

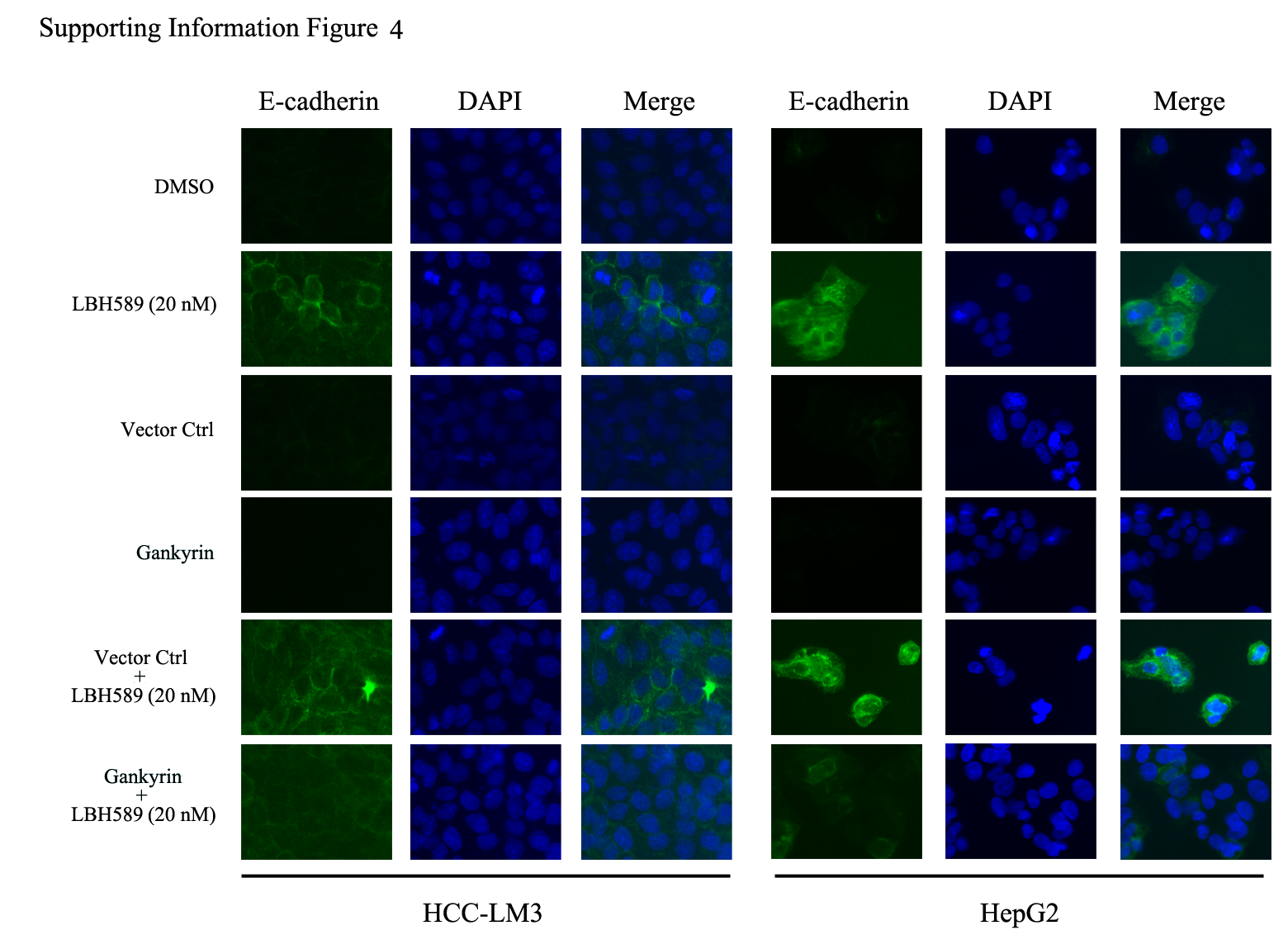

Supplement: Additional file 4: Figure S4 — Single and merged images were taken to show immunofluorescence staining of E-cadherin (green) accompanied by the cell nucleus (blue) stained by DAPI in HCC-LM3 and HepG2 cells. [file 1476-4598-12-114-S4.tiff]
